# Approaches to *in vitro* oocyte growth in domestic farm mammals: how and why?

**DOI:** 10.1590/1984-3143-AR2025-0090

**Published:** 2025-08-28

**Authors:** Valentina Lodde, Noemi Monferini, Maria Plevridi, Pritha Dey, Ludovica Donadini, Fernanda Fagali Franchi, Federica Franciosi, Alberto Maria Luciano

**Affiliations:** 1 Reproductive and Developmental Biology Laboratory – ReDBioLab, Department of Veterinary Medicine and Animal Sciences, University of Milan, Milan, Italy; 2 Center for Reproductive Biotechnology and Cryobanking, University of Milan, Milan, Italy

**Keywords:** folliculogenesis, *in vitro* folliculogenesis, oocyte culture, prematuration (pre-IVM), ovarian reserve, preantral follicles, early antral follicles

## Abstract

Unlocking the developmental potential of oocytes at various stages of folliculogenesis represents a major challenge in reproductive biology and assisted reproductive technologies. While *in vitro* maturation (IVM) of fully grown oocytes is widely applied, the vast majority of oocytes enclosed within early-stage follicles remain underutilized. This review outlines current advancements in *in vitro* culture systems designed to support oocyte growth and differentiation, with particular attention to the contributions of the authors. Key developments, mainly encompassing the bovine species, include the use of prematuration strategies to enhance the competence of oocytes retrieved from antral follicles, stepwise *in vitro* culture protocols for growing oocytes from early antral follicles, and efforts to establish defined systems for preantral follicle culture. Emerging insights into chromatin dynamics, cumulus–oocyte communication, and epigenetic regulation are shaping the design of tailored culture environments. Despite promising progress, significant challenges remain in replicating the complexity of *in vivo* folliculogenesis, particularly in non-rodent models. Addressing these challenges will be critical to expanding the oocyte pool available for reproductive and biotechnological applications, with broad implications for fertility preservation, livestock breeding, and fundamental research.

## Introduction

Understanding how to culture oocytes at different stages of their development poses a pressing challenge in reproductive biology and assisted reproductive technologies. Most oocytes in the ovary are arrested at Prophase I of meiosis and are enclosed within follicles at various stages of development, forming a highly heterogeneous population (Luciano et al., 2018; Luciano and Sirard, 2018; Sirard, 2019; Monniaux et al., 2014). However, only a small proportion of these oocytes, specifically those that have completed their growth phase, can be exploited with current reproductive technologies, either through *in vitro* maturation (IVM) or following *in vivo* maturation (Fair, 2003; Galli and Lazzari, 2008; Luciano and Sirard, 2018; Merton et al., 2003). This limitation significantly constrains the efficiency and scalability of *in vitro* embryo production systems.

IVM of “fully grown oocytes” (see below) is a well-established and widely used approach, particularly in farm animals, where immature oocytes are collected from antral follicles and matured *in vitro* (Lonergan and Fair, 2016). In contrast, *in vivo* matured oocytes are more commonly used in humans, typically following hormonal stimulation protocols such as superstimulation followed by collection before ovulation via Ovum Pick Up (OPU) (Gilchrist and Smitz, 2023; Krisher, 2022) and in the mouse model, where, depending on the experimental design and the hypothesis tested, mature oocytes are collected after superovulation from the oviduct (Menezo and Herubel, 2002). Nevertheless, neither strategy efficiently maximizes the developmental potential of fully grown oocytes, nor the resources of the ovarian reserve, as only a relatively small pool of oocytes enclosed in follicles at advanced stages of folliculogenesis are utilized in both scenarios.

Optimizing tailored multi-step culture systems for oocytes during the early stages of development, particularly those enclosed in primordial follicles, would provide invaluable tools for fundamental research, especially in non-rodent large animal models, as the bovine species, which mimics human ovarian physiology more closely (Menezo and Herubel, 2002; Sirard, 2017). Such systems are essential for advancing our understanding of basic reproductive and developmental biology. To mention just a few examples, key biological questions, such as the mechanisms of primordial follicle activation, and the role of microenvironmental factors and the extracellular matrix in supporting early follicular development, remain largely unexplored due to the lack of reliable *in vitro* experimental models (Botigelli et al., 2023; Dey et al., 2024a; Simon et al., 2020).

As already reviewed by Telfer and Andersen (Telfer and Andersen, 2021), this knowledge could have broad implications in reproductive technologies. Expanding the ability to utilize the quota of fully grown oocytes that do not progress to the blastocyst stage under standard culture conditions and exploring the differentiation of early–stage oocytes in vitro, could unlock a significant reservoir of gametes for applied *in vitro* technologies. These achievements would be particularly valuable in contexts where oocytes must be retrieved from isolated organs or recovered from endangered or sub-fertile individuals, or in research aimed at improving *in vivo* protocols by understanding the factors that affect oocyte competence. In this view, Lucy and Pohler, while addressing the “North American perspectives for cattle production and reproduction for the next 20 years” (Lucy and Pohler, 2025), have recently included the “mechanisms to stimulate the development of primordial to primary follicles and sustain development to the antral follicle stage for the purpose of improving the number of harvestable oocytes and embryos from individual cattle” among the “researchable topics and new technology with significant long-terms implications for cattle production and reproduction” (Table 3 in Lucy and Pohler, 2025). We believe it also applies to other domestic farm animals.

This review explores the advancements achieved in our laboratory, over the past two decades toward optimizing culture systems designed to enhance the growth and differentiation of bovine oocytes derived from the ovarian reserve, emphasizing both the biological rationale behind our approach and the ongoing challenges in replicating folliculogenesis *in vitro*.

## Oogenesis, folliculogenesis, and the ovarian reserve

The prerequisite for reproductive success is the formation of a fertilizable oocyte, arrested at the metaphase of the second meiotic division (MII), often referred to as “mature oocyte” or, as often indicated in rodents and humans, “egg” (Conti and Franciosi, 2018; Duncan et al., 2020). The oocyte reaches this stage through the complex steps of oogenesis, which occur in close coordination with the development of the surrounding ovarian follicle, a process termed folliculogenesis (Hyttel et al., 2010; Leung and Adashi, 2019). These steps include oocyte activation within the primordial follicle, growth, meiotic resumption, homologous chromosome segregation, emission of the first polar body, and progression to the MII stage. Completion of the second meiotic division and oogenesis occurs only after fertilization in the oviduct, involving the segregation of sister chromatids, emission of the second polar body, and formation of the female haploid pronucleus (Hyttel et al., 2010; Leung and Adashi, 2019).

In most mammalian species, the bulk of primary oocytes start the prophase I of meiosis in the fetal ovary and arrest at the diplotene stage of meiosis I, entering a prolonged resting phase, called “dictyate stage” (Hyttel et al., 2010; Leung and Adashi, 2019). During this stage, the chromosomes of the oocytes disperse, becoming indistinct and forming a loose network. Each diplotene-arrested oocyte becomes enclosed by a single layer of flattened pre-granulosa cells, forming a primordial follicle, the basic structural and functional unit of the ovary and the foundation of the ovarian reserve (Baker and Franchi, 1967; Hyttel et al., 2010; Leung and Adashi, 2019; Monniaux et al., 2014)

The dormant primordial follicles constitute a finite reserve for potential development and selection for ovulation throughout the reproductive life. Pools of primordial follicles are cyclically activated to grow, increase in size, and acquire the necessary components for further development (Hyttel et al., 2010). In their 2014 review article, Monniaux and coauthors defined these two pools of follicles as “two ovarian reserves”. The former, as the “pre-established” reserve of primordial follicles formed during fetal or early postnatal life, which serves as the long-term source of developing follicles, and the latter, as a dynamic reserve of antral follicles, responsive to gonadotropins and serving as the immediate source for ovulation and assisted reproductive technologies (Monniaux et al., 2014).

While a certain degree of activation and initial development can be observed before puberty, meiotic resumption and segregation of homologous chromosomes - the process known as oocyte maturation - occur, cyclically, only after females reach puberty (Hyttel et al., 2010; Leung and Adashi, 2019). Before being able to resume meiosis the oocytes enclosed in primordial follicles must be activated from their dormant state, decondense their chromatin, transiently re-activate transcription, and undergo an intense process of growth and cellular differentiation, until they reach their maximum size (Fair and Hyttel, 1997; Lodde et al., 2008b), in the so called “fully grown oocyte”, when transcription is again gradually silenced and the chromatin progressively compact forming the karyosphere (Bogolyubov, 2018; Luciano and Lodde, 2013; Nikolova et al., 2024). During the growth period, oocytes acquire key cytoplasmic specializations, such as the ability to produce the zona pellucida, accumulate cytoplasmic substances, undergo structural changes, and reorganize the cytoplasmic organelles. In parallel, they also undergo substantial epigenetic chromatin remodeling (Bonnet-Garnier et al., 2012; Demond et al., 2025; Edwards-Lee et al., 2025; Fair, 2003; Fair et al., 1997a, b; Fair and Hyttel, 1997; Fenner et al., 2025; Hyttel et al., 2010; Kageyama et al., 2007; Leung and Adashi, 2019; Lodde et al., 2008a, 2017). Often, the oocytes arrested at the Prophase I are referred to as “Germinal Vesicle (GV) stage oocytes”, since the nucleus (the GV) is visible under the microscope (Hyttel et al., 2010; Leung and Adashi, 2019).

As mentioned, this phase of oocyte development is closely interconnected with the ovarian follicle development, which in turn supports oocyte growth. The progression from primordial to primary follicles is characterized by the differentiation of pre-granulosa cells into a single layer of cuboidal granulosa cells and the initiation of oocyte growth. In secondary follicles, multiple layers of granulosa cells develop, and the theca cell layer begins to form around the follicle. As follicles transition to the antral (tertiary) stage, fluid-filled cavities coalesce to form the antrum, and both the granulosa and theca layers become stratified and functionally specialized (Hyttel et al., 2010; Leung and Adashi, 2019). The oocyte, still at the GV stage, continues to grow and accumulate maternal transcripts, proteins, and organelles essential for early embryonic development. Successively, when follicles develop to the antral stage (from 3 to 8 mm in the bovine species), the selection for dominance takes place and a subset of the large antral follicles (one in mono ovulatory species such as the bovine and human species) are selected for becoming dominant with the enclosed oocytes recruited to undergo maturation and be released into the fallopian tube through ovulation, upon LH surge (Dieleman et al., 2002).

Although before dominance some oocytes are already competent to develop *in vitro* into a blastocyst that can result in viable offspring after embryo transfer, additional and essential differentiation steps occur *in vivo* during subsequent follicular growth and dominance until ovulation. These processes, referred to as prematuration or capacitation, occur when a follicle is selected for dominance and are completed shortly before the LH surge, which initiates the final maturation (Dieleman et al., 2002; Hyttel et al., 1997). Coordination between oogenesis and folliculogenesis is essential: the somatic follicular environment supports oocyte development, while signals from the oocyte regulate granulosa cell function and follicle fate. This bidirectional communication ensures that only oocytes within optimally developed follicles proceed to ovulation and are competent for fertilization (Carabatsos et al., 2000; Clarke, 2022; Gilchrist et al., 2004; Jaffe and Egbert, 2017; Kidder and Vanderhyden, 2010; Matzuk et al., 2002). A schematic summary of the different stages during folliculogenesis and oogenesis is provided in Table 1.

**Table 1:** Stages of folliculogenesis and oogenesis.

**Follicular Stage**	**Follicular Characteristics**	**Oocyte Stage (Oogenesis)**	**Additional Notes**
**Primordial follicle (PMF)**	Oocyte with single layer of flattened pre-granulosa cells; quiescent reserve	Primary oocyte arrested at diplotene stage of prophase I	Low level transcription; maternal mRNA and miRNA storage; dispersed mitochondria; forms ovarian reserve
**Primary follicle (PF)**	Single layer of cuboidal granulosa cells; zona pellucida begins forming	Primary oocyte (diplotene)	Oocyte begins active growth; RNA/protein synthesis; gap junctions with granulosa cells
**Secondary follicle (SF)**	Well-defined zona; multiple granulosa layers; Granulosa cell transzonal projections extend through the zona; theca interna begins to differentiate	Primary oocyte in diplotene; active growth	Cortical granules start forming; zona pellucida thickens; accumulation of maternal transcripts and organelles
**Early antral (tertiary) follicle**	Small fluid filled cavities formation; cumulus and mural granulosa cells differentiate	Primary oocyte; still in diplotene	Oocyte near final size; competence acquisition begins; mitochondria redistribute; active bidirectional signaling between cumulus and oocyte
**Middle antral follicle**	Well-developed antrum; follicle increases in size; theca externa differentiates	Primary oocyte; still in diplotene	Active bidirectional signaling between cumulus and oocyte; cytoplasmic maturation advances; lipid and organelle redistribution; Transcriptional silencing and chromatin remodelling, oocyte “capacitation”
**Preovulatory (Graafian) follicle**	Large antrum; LH surge triggers meiosis resumption	Primary oocyte resumes meiosis I > Secondary oocyte (Metaphase I > MII)	Nuclear envelope Breakdown occurs; first polar body extruded; further cytoplasmic maturation; prepares for fertilization
**Ovulating follicle**	Follicle rupture; cumulus-oocyte complex released	Secondary oocyte arrested in Metaphase II (MII)	Cortical granules migrate beneath oolemma; zona pellucida ready for block to polyspermy
**Post-fertilization**	—	Completion of meiosis II; second polar body extruded	Triggered by sperm entry; pronuclei form; zygote stage

Due to this complex process, the number of follicles at various developmental stages, each containing an oocyte at a corresponding stage of differentiation, fluctuates throughout life and varies according to the phase of the estrous cycle.

Atresia, the physiological process by which follicles that are not destined to ovulate gradually degenerate and are reabsorbed, affects follicles at all stages, from the primordial to the preovulatory phase. Since most follicles undergo atresia rather than reaching ovulation, this process plays a key role in the continuous decline of the ovarian reserve and contributes to the progressive reduction in the number and quality of available oocytes (Dey and Luciano, 2022; Kaipia and Hsueh, 1997; Marcozzi et al., 2018; Xi et al., 2025). Although an in-depth description of the complex mechanisms leading to atresia is beyond the scope of this review, readers are encouraged to consider the early data from the group of Kruip and Dieleman, which laid the groundwork for extensive research in this field and highlighted the relationship between the stage of atresia in COCs and their developmental competence (de Loos et al., 1989, 1991; de Wit et al., 2000; Dieleman et al., 1983, 2002; Hendriksen et al., 2003; Kruip and Dieleman, 1982, 1985; Wurth and Kruip, 1992).

An extrapolation of the bovine follicle reserve at a given time in a cycling ovary is presented in an accompanying paper by our group in this same special issue (Figure 2 in Luciano et al., 2025). According to this estimate, the number of primordial, primary and secondary follicles (collectively referred to as preantral follicles) ranges between forty and sixty thousand. In contrast, the number of antral follicles is significantly lower, with early antral follicles (0.5-2 mm in diameter) enclosing growing oocytes ranges from one to two hundred and fifty, and the number of middle antral follicles (2-8 mm in diameter) ranges from twenty to thirty (Erickson, 1966; Luciano et al., 2025; Lussier et al., 1987; Modina et al., 2014; Silva-Santos et al., 2011). Moreover, published data indicate that the ovarian reserve at puberty in cattle, primarily represented by primordial follicles, averages around 84,000 in heifers, followed by a marked decline by the fourth year of life in cows (Dey et al., 2024a; Erickson, 1966; Luciano et al., 2025; Modina et al., 2014; Monferini et al., 2024; Silva-Santos et al., 2011; Telfer et al., 2023; Wallace and Kelsey, 2010). However, other factors influence early ovarian aging, as a premature decrease in the ovarian reserve occurs in both cattle and human individuals (Lin et al., 2025; Luciano et al., 2013; Modina et al., 2014; Pietroforte et al., 2024)

## The multistep approach to *in vitro* oocyte growth

**Figure 1:**
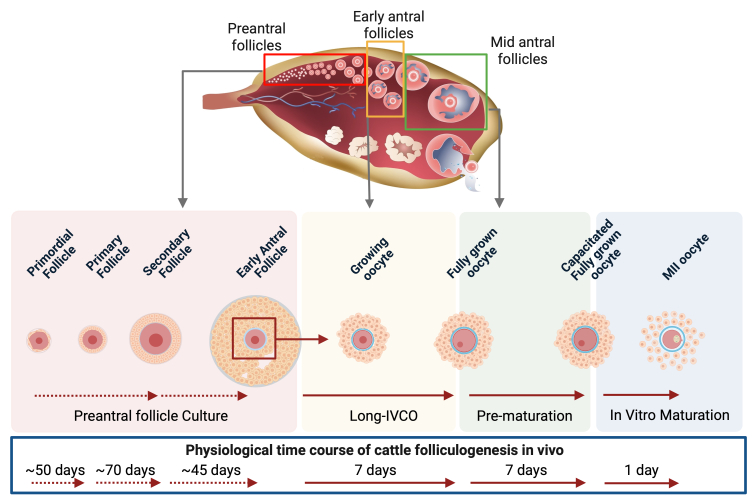
The multi-step approach to *in vitro* folliculogenesis and oocyte growth. The illustration depicts the sequential steps that should ideally be replicated *in vitro* to obtain a competent mature oocyte. Follicles and oocytes isolated from the ovary must undergo *in vitro* culture for the appropriate duration, depending on their follicle of origin and differentiation state throughout oogenesis, based on the principle that each stage of folliculogenesis has distinct biological requirements. The physiological time course shown is based on (Erickson, 1966; Fair and Lonergan, 2023; Hulshof et al., 1994; Lussier et al., 1987). Created in BioRender (2025).

Starting with the principle that each stage of folliculogenesis has distinct biological requirements, recent advances have focused on developing multi-step *in vitro* culture systems (Figure 1) to support the growth of oocytes from the primordial follicle stage to full maturation (Marcozzi et al., 2018; Morton et al., 2023; Smitz et al., 2010; Telfer and Andersen, 2021). While IVM is a well-established technique, its success largely depends on the quality and developmental competence of the oocytes, which can vary significantly due to their heterogeneity at the time of culture initiation (Luciano and Sirard, 2018; Sirard, 2019). This notion highlights the need for upstream steps that can support the controlled and physiologically relevant growth of preantral follicles, especially in non-rodent models. Ideally, each step should replicate the physiological conditions reached *in vivo* by healthy follicles (Morton et al., 2023). Clearly, this challenging goal can be achieved only through a deep understanding of the cellular and molecular determinants of the oocyte faith during the physiological process of folliculogenesis. Promising results have emerged from stepwise systems that combine activation, growth, and encapsulated culture of secondary follicles, with limited but encouraging outcomes such as the retrieval of MII oocytes and, in rare cases, blastocyst formation (Guo et al., 2024; McLaughlin et al., 2018). Nonetheless, current protocols remain inefficient, particularly in large animal models like the bovine, whose ovarian physiology and timeline of folliculogenesis parallel humans (Fair and Lonergan, 2023; Sirard, 2017).

Over the past two decades, we have addressed the challenge of developing culture systems that support the growth and differentiation of oocytes isolated from follicles at various stages of folliculogenesis. The obtained advancements include optimizing pre-IVM systems for fully grown oocytes retrieved from 2–8 mm antral follicles and culturing growing oocytes collected from 0.5-2 mm early antral follicles. Recently, we also focused on the earlier stages of development, starting with the characterization of oocytes enclosed in primordial, primary, and secondary follicles. The primary aim of these recent studies is to gather knowledge that would facilitate an “informed” setup of tailored culture systems for each class of preantral follicles in cattle.

## Prematuration (pre-IVM) as a strategy to culture oocytes isolated from middle antral follicles and enhance their competence: rationale and development of a tailored approach

The variable outcomes of IVM stem partly from its inability to replicate the complexity of the *in vivo* follicular environment (De Vos et al., 2021; Fair and Lonergan, 2023; Hendriksen et al., 2000; Lonergan and Fair, 2016; Luciano et al., 2018). *In vivo*, oocytes acquire meiotic and developmental competence during the final stages of folliculogenesis, influenced by a specific hormonal milieu, among which, a low, sustained levels of follicle-stimulating hormone (FSH) plays a pivotal role (Buratini et al., 2022; Conti and Franciosi, 2018; Dieleman et al., 2002; Hendriksen et al., 2000; Morton et al., 2023). Experimental evidence has demonstrated that, in vitro, physiological level of FSH supports chromatin remodeling and maintains communication between oocytes and cumulus cells (CCs) (Franciosi et al., 2009, 2014; Lodde et al., 2013; Luciano et al., 2011; Luvoni et al., 2006), likely promoting, through a cAMP-mediated process (Guixue et al., 2001; Luciano et al., 1999, 2004), the synchrony of nuclear and cytoplasmic maturation, both of which are essential for successful fertilization and embryonic development.

However, meiosis resumes spontaneously when cumulus-oocyte complexes (COCs) are isolated from middle antral follicles (Pincus and Enzmann, 1935; Jaffe and Egbert, 2017). The abrupt removal interrupts the final differentiation process, forcing oocytes to proceed through meiosis regardless of their differentiation stage (Luciano et al., 2018). To address this limitation, prematuration, or “pre-IVM”, strategies have been developed by several groups across different species, including humans (Dieci et al., 2013; Franciosi et al., 2014; Gilchrist et al., 2024; Hendriksen et al., 2000; Lodde et al., 2019; Luciano et al., 2021, 2023; Richani and Gilchrist, 2022; Smitz et al., 2025; Albuz et al., 2010). Pre-IVM is primarily designed to support the so-called “capacitation” phase of the oocyte, described by Poul Hyttel in 1997 (Hyttel et al., 1997) and to restore the physiological synchrony between meiotic resumption and cytoplasmic readiness (Hyttel et al., 1997). Indeed, these protocols aim overall to prolong meiotic arrest *in vitro*, typically through cAMP modulation, allowing oocytes additional time to complete cytoplasmic and molecular maturation.

Building on decades of research into the molecular mechanisms governing meiotic arrest and resumption (Albertini et al., 2001; Eppig, 2001; Gilchrist, 2011; Gilchrist et al., 2016; Jaffe and Egbert, 2017; Luciano et al., 2004; Sánchez and Smitz, 2012; Tsafriri et al., 1996), our group developed a pre-IVM culture systems in cattle based on two key principles: the necessity of preserving the natural meiotic arrest, and the importance of accounting for the intrinsic heterogeneity of oocytes retrieved from antral follicles (Luciano and Sirard, 2018). Our culture system is designed to maintain high intra-oocyte cAMP levels, preserve gap junction-mediated communication with CCs, and support orderly chromatin compaction, employing mild FSH stimulation to sustain intercellular communication without triggering premature meiotic resumption (Franciosi et al., 2014; Lodde et al., 2013; Luciano et al., 2011; Soares et al., 2017). This extended pre-IVM window is intended to support critical events such as transcript accumulation/maturation and organelle reorganization within the oocyte, both of which are vital for full developmental competence (Luciano et al., 2021).

A pivotal aspect of our approach is recognizing that oocytes within the same follicular cohort, as assessed by the follicle diameter, are not developmentally equivalent (Dieci et al., 2016). To manage this heterogeneity, we leveraged large-scale chromatin configuration as a reliable morphological marker of oocyte differentiation. Specifically, we categorized oocytes into GV0 to GV3 stages, reflecting increasing levels of chromatin compaction and, generally, higher competence (Lodde et al., 2007, 2008b; Luciano and Lodde, 2013). Among these, GV1-stage oocytes, defined by intermediate chromatin compaction, were identified as the most responsive to pre-IVM treatment (Dieci et al., 2016).

Experimental data indeed demonstrated that COC classified as class 1, according to (Blondin and Sirard, 1995), are enriched in GV1 oocytes and significantly benefit from the Pre-IVM treatment, showing improved developmental potential up to the blastocyst stage. In contrast, class 2 and 3 COCs, which do not contain GV1 oocytes and are instead enriched in GV2 and GV3 oocytes, were unresponsive to Pre-IVM and exhibited reduced developmental outcomes when subjected to prolonged culture (Dieci et al., 2016).

These findings were further supported by transcriptomic profiling of CCs, which revealed that gene expression patterns are closely linked to the chromatin status of the enclosed oocyte. CCs associated with GV1 oocytes express transcripts related to survival and competence, while those surrounding GV3 oocytes exhibit molecular signatures of stress and apoptosis, including upregulation of caspase activity (Dieci et al., 2016). This molecular evidence reinforces the importance of accurate oocyte staging and the selective application of pre-IVM. Further attempts to modulate the pre-IVM system, such as alternative strategies to block meiotic resumption using Natriuretic Peptide type C (NPPC) and/or adjusting the hormonal composition of the culture medium, have resulted in improvements of blastocyst quality, but not in an increase in blastocyst formation rates (Franciosi et al., 2014; Soares et al., 2017). Since these treatments were applied to a heterogeneous population of COCs collected from medium-sized antral follicles, it remains to be determined whether they may be particularly beneficial for GV1 oocytes. This also raises an unresolved question in reproductive biology: whether, and to what extent, early signs of atresia, such as those observed in COCs enclosing a GV3-stage oocyte, can be rescued *in vitro*.

Our findings were further supported by a subsequent *in vivo* study, where we demonstrated that synchronizing oocyte nuclear maturation to enrich the population of GV2-stage oocytes can significantly enhance the efficiency of *in vitro* embryo production in Holstein cows. We obtained a highly homogeneous cohort of oocytes by applying a mild FSH synchronization protocol combined with follicular aspiration, with 83% at the GV2-stage. This enrichment was associated with a marked improvement in embryonic development, as evidenced by a significantly higher blastocyst rate and a tendency toward an increased proportion of embryos classified as suitable for cryopreservation (Soares et al., 2020). These results underscore the importance of controlling oocyte nuclear maturity at the time of collection, highlighting the GV2-stage as a key window of developmental competence. Synchronizing oocytes to this stage may represent a practical strategy to optimize embryo yield and quality in IVP programs.

In summary, our prematuration approach is grounded in a mechanistic understanding of oocyte biology and tailored to accommodate the inherent developmental variability of oocytes aspirated from antral follicles. By identifying and selectively treating oocytes at the GV1 stage, we enhance the efficiency of assisted reproductive technologies and avoid the pitfalls of indiscriminate *in vitro* culture. These insights provide a rationale for personalized pre-IVM strategies to optimize oocyte developmental competence *in vitro*.

## Development of a culture system for bovine oocytes isolated from early antral follicles: a stepwise approach guided by oocyte biology

Our group has progressively developed and optimized an *in vitro* culture (IVCO) system for bovine oocytes isolated from early antral follicles (EAFs) to recapitulate the *in vivo* environment that supports oocyte growth and acquisition of meiotic and developmental competence. The oocytes enclosed in these follicles remain in their growing phase and are unable to resume meiosis spontaneously when isolated (Fair, 2003; Fair et al., 1997a, b; Fair and Hyttel, 1997; Lodde et al., 2007, 2008b). Under fluorescence microscopy, the chromatin of these oocytes displays a filamentous pattern enclosed in the GV, a configuration classified as GV0, showing low levels of global DNA methylation and histone acetylation compared to fully grown oocytes with higher degree of chromatin compaction (Lodde et al., 2007, 2008a, 2009, 2017; Saraiva et al., 2025). Importantly, GV0 oocytes are fully coupled with their surrounding CCs through open gap junctions (Lodde et al., 2007; Luciano and Lodde, 2013), a prerequisite for establishing a proper *in vitro* culture to support further differentiation of these growing oocytes (Luciano et al., 2014; Luciano and Lodde, 2013).

Building on previous evidence correlating cAMP levels with junctional coupling (Luciano et al., 2004; Modina et al., 2001), our foundational work demonstrated the central role of cumulus–oocyte communication via gap junctions in orchestrating chromatin remodeling and transcriptional regulation (Luciano et al., 2011). A 24-hour IVCO protocol utilizing serum-free TCM-199 medium supplemented with physiological doses of recombinant FSH and cilostamide (a PDE3 inhibitor that preserves intra-oocyte cAMP) supported the maintenance of oocyte-cumulus cells coupling, oocyte growth, and the GV0-GV1 chromatin transition, enhancing meiotic progression and developmental outcomes (Luciano et al., 2011). IVCO system was later refined, identifying zinc as a key regulator of nuclear and epigenetic maturation. Zinc sulfate supplementation during IVCO supported oocyte growth, preserved transcriptional activity during early chromatin remodeling, and modulated global DNA methylation patterns, highlighting zinc’s critical involvement in regulating gene expression and epigenetic plasticity during the GV transition (Lodde et al., 2020).

Recently, a refined protocol, termed Long IVCO (L-IVCO), that more closely mimics the physiological environment in which GV0 oocytes grow *in vivo*, significantly advanced the success of the culture (Barros et al., 2020; Garcia Barros et al., 2023). In this work, we systematically evaluated the effects of adding to the IVCO medium steroid hormones, such as estradiol (E2), progesterone (P4), and testosterone (T), at the physiological concentrations found in the corresponding follicle stage (Aardema et al., 2013; Beg et al., 2002; Castilho et al., 2019; Dieleman et al., 1983; Endo et al., 2013; Fortune and Hansel, 1985; Henderson et al., 1982; Kruip and Dieleman, 1985; Makita and Miyano, 2015; Modina et al., 2007; Sakaguchi et al., 2019; Tessaro et al., 2011). Furthermore, we extended the culture duration to 5 days, aligning it with the duration of the corresponding step of bovine folliculogenesis (Erickson, 1966; Fair and Lonergan, 2023; Hulshof et al., 1994; Lussier et al., 1987) and incorporated several optimized components: collagen-coated plates to support COC morphology differentiation, zinc sulfate to support the transcriptional activity, and polyvinylpyrrolidone to modify viscosity and promote a 3D-like organization (Alam et al., 2018; Hirao et al., 2004). The L-IVCO system preserved cumulus–oocyte complex architecture, supported progressive chromatin maturation up to the GV3 stage, transcriptional silencing, oocyte growth, and ultimately improved IVM rates, enhancing cumulus expansion and blastocyst development following IVF (Barros et al., 2020; Garcia Barros et al., 2023).

Importantly, the L-IVCO system has recently been successfully translated in sheep, achieving essential milestones and demonstrating the feasibility of producing developmentally competent oocytes from early antral follicles in this species (Ebrahimi et al., 2024a, b, 2025). L-IVCO can support growth and enable oocyte meiotic maturation, showcasing promising outcomes regarding cumulus expansion, mitochondrial activity, and reduced oxidative stress. Our group is also focused on translating the protocol to other farm animals, such as the horse (Unpublished data).

Although the L-IVCO system has significantly enhanced the ability to culture growing oocytes *in vitro*, oocyte competence remains suboptimal. Future efforts will concentrate on integrating dynamic culture systems, real-time monitoring of metabolic and epigenetic markers, and refining the somatic microenvironment. Nevertheless, this model offers a robust and manageable system to enlarge the gamete pool and investigate oocyte developmental biology in physiological as well as environmentally challenged conditions.

A key biological question to be answered to enhance the system is a better understanding of the mechanisms regulating transcription and maintaining the cross-talk between oocytes and CCs. This goal is accomplished *in vivo* through paracrine and gap junction-mediated processes, and its decoding would allow for the modulation of the media composition to support a more prolonged culture, extending beyond the current 5 days established in our protocol.

## Approaches to preantral follicle culture

Recapitulating early folliculogenesis outside the ovarian environment must adhere to the overarching principle of creating a biomimetic system that supports the survival, growth, and differentiation of individual follicle stages: primordial, primary, and secondary. As outlined in our recent review paper, tailoring the culture conditions to the unique metabolic, structural, and signaling needs of each follicle type is critical to this endeavor, requiring precise control over factors such as oxygen tension, extracellular matrix composition, and growth factor supplementation (Dey et al., 2024b). This information is challenging to obtain in a system where follicles are commonly cultured “in situ”, i.e., within small ovarian cortex fragments, due to difficulties in tracking the fate of individual follicle, and thereby the culture of isolated follicles would be more informative. Ideally, the culture of isolated follicles would provide more information. However, a major limitation lies in the intrinsic fragility of primordial follicles once isolated, as they are highly sensitive to mechanical and enzymatic stress and prone to programmed cell death outside their native stromal context (Dey et al., 2025). This issue, complicated by survivorship bias in culture experiments and the lack of standardized follicle quantification and viability assessment methods, significantly hinders the reproducibility and translational success of preantral follicle culture systems in large mammals. Due to these limitations, current literature on *in vitro* culture of preantral follicles is jeopardized by significant methodological variability and lack of standardization (Dey et al., 2024a, b; Simon et al., 2020).

Over the past 30 years, *in vitro* culture of preantral follicles has advanced significantly in the murine model (Dey et al., 2024b; Simon et al., 2020), beginning with the culture of primordial follicles to obtain newborns (Eppig and O’Brien, 1996; O'Brien et al., 2003) and utilizing gene editing to study the mechanisms of folliculogenesis (Li et al., 2010; Nagamatsu et al., 2019; Shah et al., 2018; Zheng et al., 2023). In contrast, results in higher-mammalian species are limited and experimental (Frost and Gilchrist, 2024; Telfer and Andersen, 2021). Two of the most relevant results were obtained in humans, where a few mature oocytes (MII) have been derived from primordial follicles (McLaughlin et al., 2018) and a single blastocyst has been developed from secondary follicle culture (Guo et al., 2024). These sparse findings highlight the profound interspecies differences, particularly between mice and humans (Campbell et al., 2003; Ménézo and Hérubel, 2002; Sirard, 2017). Like other higher-order mammals such as cattle, humans experience a longer duration of folliculogenesis (Denicol, 2024; Dey et al., 2024b; Monniaux et al., 2014), necessitating a more extended and tightly controlled *in vitro* culture system. In mice, the total culture time from ovary culture to the MII stage was 23 days, divided into multiple steps, resulting in 59 newborns (O'Brien et al., 2003). For humans, however, adopting a multistep procedure over 27 days, starting from 160 ovarian cortical slices, resulted in only 9 oocytes showing abnormal polar bodies (McLaughlin et al., 2018). Improved outcomes have been reported in a recent study where a protocol was developed for human follicle culture starting from the secondary stage. By extending the culture period to 4–6 weeks, this approach yielded 10/71 and 4/70 MII oocytes when cultured with or without the addition of neurotrophic factor 4, respectively, and resulted in one blastocyst (Guo et al., 2024). The culture period in this protocol represents approximately half the physiological duration of folliculogenesis from the secondary to the mature stage *in vivo*, estimated at 85 days (Gougeon, 2004), suggesting that optimizing the timing of *in vitro* culture may enhance the developmental outcomes. However, extending the culture period must be finely regulated to sustain the growth and survival of the follicles. Due to its physiological similarities to humans, cattle may serve as a valuable experimental model for developing *in vitro* systems for human folliculogenesis. So far, results in bovine culture of preantral follicles indicate a highly divergent timeline, ranging from 4 to 32 days of follicle culture starting from the cortical strips (Braw-Tal and Yossefi, 1997; Derrar et al., 2000; Gigli et al., 2006; Telfer et al., 2000; Wandji et al., 1996) or the secondary stage (Araújo et al., 2015; Gutierrez et al., 2000; McLaughlin et al., 2010; Rossetto et al., 2013; Taketsuru et al., 2011). Until now, results have shown reduced viability and low stage transition of healthy follicles.

We believe that the only way to fulfill *in vitro* folliculogenesis is through the accurate analysis of the cellular and molecular characteristics of the follicles at different stages of their development. This analysis is crucial for informing the scientific community about the requirements (biochemical signals, developmental timing, cell-cell communication, biomechanical cues) of each developmental stage. By studying the mechanisms that guide follicle growth, we can establish differentiation markers and define the appropriate culture environment. With this in mind, over the past few years, we have focused our research efforts on characterizing the population of preantral follicles at different developmental stages (primordial, primary, and secondary) in the bovine model. We established a high-yield, enzyme-free mechanical isolation protocol that preserves follicle morphology and viability, enabling the recovery of hundreds of intact follicles from minimal cortical tissue (Dey et al., 2024a; Monferini et al., 2024). This method provides a reproducible strategy suitable for fertility preservation studies. Building on this foundation, we developed a defined, serum-free culture system tailored to primordial follicle needs, allowing for short-term survival and experimental manipulation (Dey et al., 2025). Using transcriptomic profiling, we identified ferroptosis as a primary response mechanism of follicle attrition *in vitro*. We demonstrated that glycine supplementation can mitigate oxidative stress and prolong follicle viability (Dey et al., 2025). Together, these advances provide a robust and scalable platform for mechanistic studies and the future refinement of long-term culture systems aimed at supporting complete folliculogenesis from the dormant pool. Studies are in progress to further characterize the population of primary and secondary follicles in the bovine model. Additional information on the ongoing studies in our laboratory is presented in the accompanying paper in this special issue (Luciano et al., 2025).

## Conclusions and perspectives

Despite the considerable progress in developing *in vitro* systems to support oocyte growth and differentiation in large mammals, current outcomes remain suboptimal and reflect the gaps in our understanding of the fundamental mechanisms governing folliculogenesis and oogenesis. A key concept emerging from both our experience and the literature is that each stage of follicular development has specific physiological requirements, including growth factor milieu, cell-cell communication dynamics, and metabolic activity, which must be carefully recapitulated *in vitro*. Therefore, culture media should be formulated in a stage-specific manner that reflects the natural ovarian environment with strategies grounded in biological rationale.

This need is particularly evident in the field of antioxidant supplementation, which has, in some cases, been applied indiscriminately. While oxidative stress is a recognized threat to oocyte and embryo viability *in vitro*, recent data highlight that the efficacy of antioxidants is highly context-dependent and dose-sensitive (Naspinska et al., 2023). Although compounds like melatonin, cysteamine, and lipoic acid have shown benefits under certain conditions (Canel et al., 2018; Fabra et al., 2020; Lodde et al., 2021; Rodrigues-Cunha et al., 2016), the inclusion of non-validated molecules or plant-derived extracts (e.g., carvacrol, eugenol, Aloe vera) often lacks mechanistic justification (Azevedo et al., 2022; Morais et al., 2023; Silva et al., 2022). Furthermore, supraphysiological concentrations can lead to “reductive stress,” disrupting key redox-sensitive pathways and mitochondrial function (Gameiro et al., 2013; Gao and Wolin, 2008; Giorgio et al., 2007; Ho et al., 2017; Kannan et al., 2013). This has been demonstrated with compounds like MitoQ, where high doses impair developmental competence despite their antioxidant labeling (Marei et al., 2024). A more promising approach may be to support the oocyte’s intrinsic strategies for regulating ROS, such as modulating mitochondrial activity to reduce ROS generation at the source, as demonstrated in human and *Xenopus laevis* oocytes (Rodríguez-Nuevo et al., 2022). Although this approach remains technically demanding in large animal models, it offers a conceptually elegant alternative to the addition of external antioxidants.

Notably, the developmental competence of oocytes appears irreversibly compromised once early signs of atresia emerge, as evidenced by the marginal gains in blastocyst formation despite intense research efforts. This observation highlights a critical conceptual shift: successful *in vitro* growth must start from oocytes that are not yet committed to atresia. A central unanswered question is whether atresia is reversible at its earliest stages, and which molecular pathways dictate this fate. Addressing these gaps of knowledge, especially in understanding the early events that govern oocyte-somatic cell communication, transcriptional control, and survival signaling, will be key to refining culture conditions, extending culture periods, and ultimately unlocking the full potential of the ovarian reserve.

## Data Availability

No research data was used.
